# Intra-individual variability and continuity of action and perception measures in infants

**DOI:** 10.3389/fpsyg.2015.00327

**Published:** 2015-03-25

**Authors:** Anja Gampe, Anne Keitel, Moritz M. Daum

**Affiliations:** ^1^Department of Psychology, University of Zürich, Zürich, Switzerland; ^2^Centre for Cognitive Neuroimaging, Institute of Neuroscience and Psychology, University of Glasgow, Glasgow, UK

**Keywords:** action, perception, infancy, variability, continuity

## Abstract

The development of action and perception, and their relation in infancy is a central research area in socio-cognitive sciences. In this Perspective Article, we focus on the developmental variability and continuity of action and perception. At group level, these skills have been shown to consistently improve with age. We would like to raise awareness for the issue that, at individual level, development might be subject to more variable changes. We present data from a longitudinal study on the perception and production of contralateral reaching skills of infants aged 7, 8, 9, and 12 months. Our findings suggest that individual development does not increase linearly for action or for perception, but instead changes dynamically. These non-continuous changes substantially affect the relation between action and perception at each measuring point and the respective direction of causality. This suggests that research on the development of action and perception and their interrelations needs to take into account individual variability and continuity more progressively.

## Action and Perception in Development

Everyday social interactions involve the production of one’s own actions and the perception of actions performed by others (henceforth referred to as action and perception). In the last two decades, a great amount of research has shown that action and perception are mutually related (e.g., Prinz, 1997) and focused on the particular influence of action on perception and vice versa. It has been shown that the perception of others’ actions is improved in those with their own action abilities (e.g., Hamilton et al., 2004; Calvo-Merino et al., 2005), and that observing others’ actions influences subsequent own action execution (e.g., Craighero et al., 2002; Kilner et al., 2003). This relation between action and perception is especially interesting from a developmental perspective, because during the first months of life infants are about to develop both action and perception skills. It is thus considered possible to disentangle the relative contributions of action and perception for the development of a mutual link. However, there is an ongoing debate about the temporal order of action and perception development, thus whether infants have to be able to perform an action before they can understand it or vice versa (Hauf et al., 2007). Concerning the mutual relation, some studies suggest that a link between action and perception is already present early in life (e.g., Nyström, 2008; van Elk et al., 2008; Kanakogi and Itakura, 2011; Ambrosini et al., 2013). For example, Daum et al. (2011) have found a correspondence between 6-month-old infants’ grasping skills (palmar vs. thumb opposition) and their differentiation between expected and unexpected grasping actions (longer looking times toward incongruent grasping actions, i.e., large hand aperture for small objects and vice versa). Studies measuring anticipatory gaze have found that between 4 and 10 months of age, one-handed grasping was correlated with gaze latency toward the goal of human grasping actions (Kanakogi and Itakura, 2011). Melzer et al. (2012) used a combined perception-action task to investigate the development of contralateral reaching in infants at 6 and 12 months. In the perception task, videos of either contralaterally or ipsilaterally grasped and transported objects were presented and anticipatory gaze behavior was analyzed. In the action task, infants’ ipsi- and contralateral reaching behavior toward toys was analyzed to see how often they already reached contralaterally. At 12 months, infants’ anticipation of contralateral actions was correlated with their contralateral reaching skills (Melzer et al., 2012). This correlation was not yet evident in 6-month-old children. The above-mentioned studies suggest a link between action and perception in infancy, although the occurrence varies with respect to age and the particular action. Importantly, the state of evidence is not homogenous. When investigating different abilities at different measuring points, different conclusions on the strength and the causality between action and perception are claimed. Some authors suggest that there is an immediate link between action and perception as soon as an action can be produced (Sommerville et al., 2005; Kanakogi and Itakura, 2011; Ambrosini et al., 2013). Others suggest that active experience with an action is necessary before it is linked to perception (cf. Cannon et al., 2012; Melzer et al., 2012). And still other studies report that perception develops to some extent independently of action abilities (Gergely et al., 1995; Hofstadter and Reznick, 1996; Hofer et al., 2005; Biro and Leslie, 2007). Sometimes even the same lab shows a link between action and perception in one study (grasping; Bakker et al., 2014) but not in another (pointing; Gredebäck et al., 2010).

But where do these contradictory results derive from? Potential factors include the designs used, the abilities looked at, the measures calculated, or the age group investigated. In this Perspective Article, we argue that one important but previously neglected factor is the nature of developmental processes: Often, the implicit assumption is that development is more or less continuous. But do abilities really improve steadily and linearly? There is much evidence that, at group level, action and perception skills consistently improve with age (Van der Fits et al., 1999; Hofer et al., 2005; Falck-Ytter et al., 2006; Kanakogi and Itakura, 2011; Ambrosini et al., 2013; Keitel et al., 2013, 2014; Gampe and Daum, 2014). The group level results of Melzer et al. (2012) showed, for example, both an increase in contralateral reaching and an increase in anticipations of contralateral movements between 6 and 12 months. But less is known about the particular shape of developmental trajectories at the individual level. For example, dynamic systems theory suggests that individual development might look quite different from average group development (Thelen and Smith, 2007). According to this approach, abilities self-organize and adapt to their surroundings dynamically (Smith and Thelen, 1993). Behavior emerges as a result of the relationships between abilities. Importantly, abilities are not linearly bound, which means that a small change in one single ability can result in a transformation of the whole system.

The only possibility to investigate individual development is to collect longitudinal data on action and perception skills in infants, and correlate these measures over developmental time. If individual development is linear, good performance at one measuring point should surely entail good performance at another. Such a consistency should also result in high correlations for action and/or perception measures at different measuring points within and between domains. In this Perspective Article, we argue that this is often not the case, and present supporting data from one longitudinal study.

## Longitudinal Data on Action and Perception Development

**FIGURE 1:**
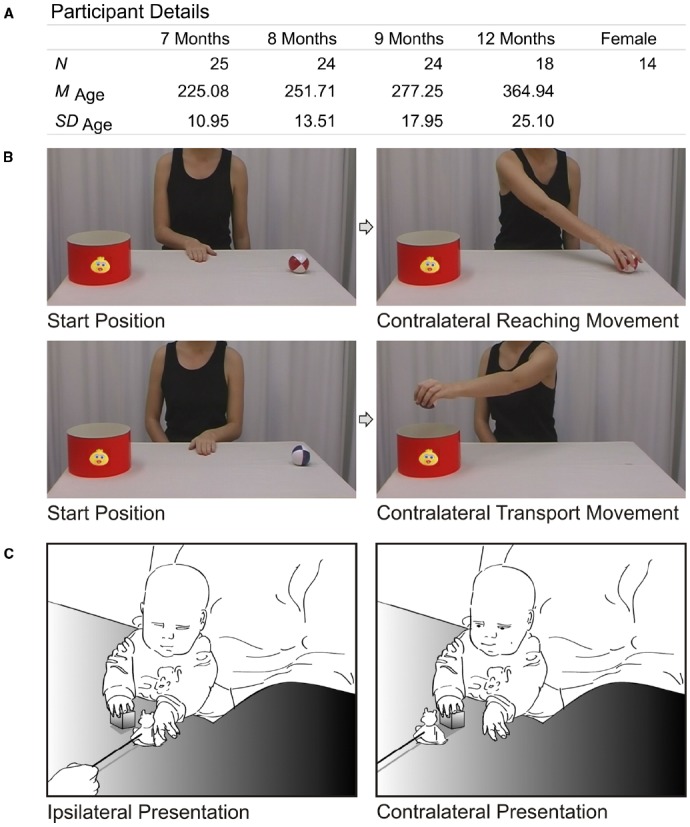
**Methods. (A)** Participant details, including number of participants per measuring point, mean age in days (and standard deviation), and number of females in the sample. **(B)** Screen shots of the perception task. Upper row illustrates a trial with a contralateral reaching movement (and, therefore, an ipsilateral transport movement, not shown). Lower row illustrates a trial with a contralateral transport movement (and preceding ipsilateral reaching movement, not shown). While observing the actions, infants’ eye movements were recorded using an eyetracker (SR Research Eyelink Plus, 500 Hz, monocular). As perception measure, we calculated the anticipation frequency for contralateral movements, that is, the number of trials in which the gaze arrived at goal areas (i.e., ball AOI and bucket AOI) prior to the model’s hand, divided by all actions that were perceived. We used the same criteria for analysis as in the original study (Melzer et al., 2012). **(C)** Illustration of the action task. We presented similar toys on sticks as Melzer et al. (2012), while the child held a small cube in one hand. The toys were either presented to the empty hand of the child (to elicit an ipsilateral reaction) or to the occupied hand (to elicit a contralateral reaction with the empty hand). As a measure of action, we calculated the ratio between the performed contralateral grasping movements (interrater-reliability κ = 0.93) and presented contralateral trials: *N*_contralateral_grasped_/*N*_contralateral_presented._ Licenses for re-used figures from Melzer et al. (2012) have been obtained.

To substantiate our argument, we tested the intra-individual variability and continuity of perception and action in infancy. To this end, we tested 25 infants longitudinally at 7, 8, 9, and 12 months of age (see Figure 1A for details), using the action-perception paradigm developed by Melzer et al. (2012), in which perception and production of contralateral grasping movements were measured.

In the perception task (see Figure 1B for details), children observed videos of an actor grasping a ball (either ipsilaterally or contralaterally) and transporting it into a bucket (either contralaterally or ipsilaterally). The frequency of anticipatory gaze shifts toward the goal of contralateral movements was used as a performance measure. In the action task (see Figure 1C for details), the children’s ability to reach contralaterally was tested. The frequency of contralateral responses produced toward a presented toy was used as a measure of action. Action and perception measures were both expressed in per cent, which makes them easily comparable.

**FIGURE 2:**
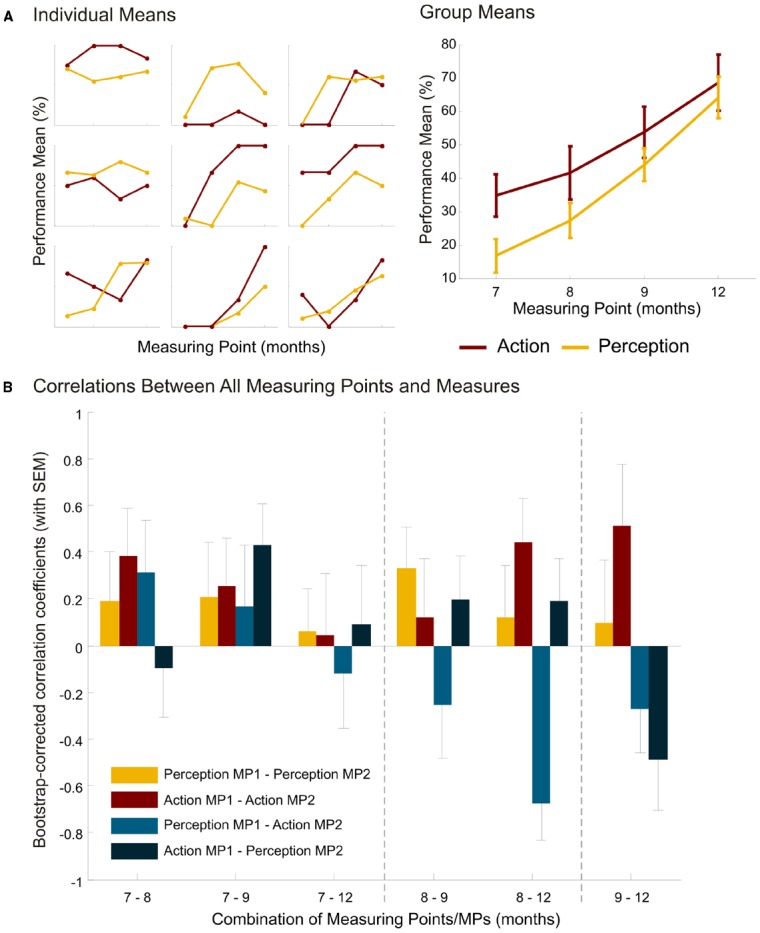
**Results. (A)** Mean performance for action and perception (in %) of nine individual children and of the group with standard error of the mean at the different measuring points (7, 8, 9, and 12 months of age). Individual data displayed is of the children who provided action and perception measures at all measuring points. Although the number of participants was initially 25, only 18 children participated at age 12 months. Of those 18 who participated at all measuring points, only 9 consistently provided performance measures in both tasks. Note that the y-axis for each individual plot is scaled from 0 to 100%. **(B)** Bootstrap-corrected correlation coefficients (with SEM) for perception, action, perception-action and action-perception between measuring points (MPs) 7–8, 7–9, 7–12, 8–9, 8–12, and 9–12 months.

At group level, we found similar action and perception abilities to those in the original study (see Figure 2A for individual and group means). The anticipation frequency increased from *M*_7 months_ = 16.8 ± 22.4% (± SD) to *M*_12 months_ = 64.2 ± 28.6% (Melzer et al., 2012: *M*_6 months_ = 19.1 ± 3.2%, *M*_12 months_ = 61.8 ± 3.8%). The frequency of contralateral reaching increased from *M*_7 months_ = 18.2 ± 14.8% to *M*_12 months_ = 34.3 ± 18.1% (Melzer et al., 2012: *M*_6 months_ = 18.9 ± 15.9% to *M*_12 months_ = 30.7 ± 15.4%).

However, we were interested in a systematic evaluation of the continuity of action and perception measures at group level (linear regression) and at individual level (correlations). To this end, we ran linear regression analyses for action and perception with age in days as the between-subject factor. Performance increased linearly for action, *R*^2^ = 0.09, *p* = 0.007, and for perception, *R*^2^ = 0.25, *p* < 0.001. The regression coefficients for action and perception differed significantly, *t* = 2.15, *p* = 0.03, suggesting that age is a stronger predictor for perception than for action. A steeper increase in perception abilities than in action abilities is thus evident. *Post hoc* Bonferroni-corrected *t*-tests with all infants revealed that, for action, performance differed significantly between 7 and 12 months (*p* = 0.003). For perception, performance differed between the following age groups: 7–9: *p* = 0.002; 7–12: *p* < 0.001; 8–9: *p* = 0.009; 8–12: *p* < 0.001, but not for 7–8 and 9–12 months.

In a second step, we looked at the correlations within a performance measure between measuring points to see whether the abilities also increase linearly at individual level (Figure 2B, yellow and red bars). More precisely, we correlated perception at all different measuring points, and action at all different measuring points. If individual development was linear, there should be significant correlations between different measuring points, with the highest correlation coefficients at adjacent measuring points. To get a better estimation of the correlation, we bootstrapped the pairwise correlations and calculated the correlation bias and the standard error of the mean correlation coefficient. The p-values were corrected for multiple testing (FDR correction) and showed that none of the perception abilities (all *p* > 0.67) and none of the action abilities (all *p* > 0.24) were correlated with the same ability at another measuring point.

A further analysis targeted questions of the temporal order of action and perception. Are we able to perform actions ourselves only after having understood other people’s actions, or do we need own action abilities for observational understanding of others? We again calculated bivariate, bootstrap-corrected correlations with FDR-corrected p-values between perception ability at one measuring point and action ability at another measuring point and vice versa (Figure 2B, light and dark blue bars). Correlations yielded no significance for action predicting perception at any measuring point (all *p* > 0.20). Perception at 8 months negatively predicted action at 12 months (*r* = –0.659, *p* < 0.05); and no other significant predictions from perception to action (all other *p* > 0.48).

Finally we looked at the relations between action and perception at one measuring point, as was done in the original study. We did not find a correlation between action and perception measures at 12 months of age, nor at any other age tested. The highest correlation we found is *r* = 0.33 at 7 months of age, which did not reach significance (*p* = 0.16).

The longitudinal data presented illustrate two points: First, action and perception increase linearly at group level but not at individual level. Second, correlations between action and perception within and between measuring points are unstable and transient. Not one level of abilities relates to its ability at a later stage, although abilities at group level increase steadily. The relations between the domains are of different strengths at different points in time and between points in time. Together, these findings suggest that individual development does not take place linearly, but might depend on various interactions of specific abilities within the child, which affects performance at any given time.

## Action and Perception Development within a Dynamic System

This idea is congruent with the view that abilities self-organize and adapt to their surroundings dynamically as proposed, for example, by the dynamic systems approach (Smith and Thelen, 1993). When looking at the longitudinal results presented above, it appears that, in contrast to group level, at individual level perception and action do not develop in a continuous manner, but rather in developmental trajectories that differ greatly between individuals (for a discussion of a variety of individual developmental trajectories, see, e.g., Adolph et al., 2008). The present findings add to this knowledge that, resulting from these individual differences, the relation between perception and action is not one of continuous stability but also subject to fluctuations over age. Transferred to system dynamics, this means that action and perception abilities are themselves the result of relationships with other abilities that can change at any moment. How each ability develops over time therefore depends on various interactions with other abilities within each infant. As a consequence, no individual correlation was found within one domain over the measuring points. Some of the infants improved in comparison to the last measure, while others remained constant or declined. At group level, a linear increase can be observed because performance increases in more infants than it decreases. And even within action and perception abilities a small, but critical change in one sub-system can cause the whole system to shift, resulting in a new action or perception behavior. This way, the strength of the relation between action and perception and predictive power in different measuring points varied enormously.

## Consequences and Possible Solutions

The most important message of the above findings and theoretical considerations is that a cautious interpretation should be made when relations and especially temporal order of action and perception are investigated in infants. Unsteady individual development can make a replication of results difficult, which is evident in the heterogeneity of previous findings, as well as in the discrepancy between the current data and Melzer et al. (2012). Although we found the same level of abilities in action and perception at group level, we were unable to replicate the interrelation between them. One reason might be the difference in design (cross-sectional vs. longitudinal), another might be the sample size for infants who provided data in action and perception measures at each measuring point (*N*_7 months_ = 20; *N*_8 months_ = 24; *N*_9 months_ = 20; *N*_12 months_ = 14 vs. *N* = 24 in the original study). But as we have replicated the results at group level, it also seems plausible that system dynamics might account for the missing relation. Abilities in dynamic systems are unstable and unpredictable in transition phases (Lewis, 2000). As a result, some studies will find no relations while others might see incidental relations. Non-linear individual development could, consequently, also cause non-linear results at group level (van der Maas and Molenaar, 1992; van Geert, 1994). This is rarely found in published data, although this could be due to the fact that researchers usually expect continuous results, and do not attempt to publish erratic data (but see Keitel and Daum, 2015). Answers to simple questions of temporal order or functional relations between action and perception cannot therefore be unidimensional but depend on the age group chosen, the distance between measures and the domains and abilities looked at.

There are some methodological precautions one could take to ensure that an interpretation of findings is reliable, at least to some extent. For example, sample size should be large enough to accurately reflect the population, going beyond the 10–12 children per group sometimes reported (Gredebäck et al., 2010; Kanakogi and Itakura, 2011; Ambrosini et al., 2013). A large number of trials helps to yield the most reliable results, although this might not always be easy to achieve with infants. Collecting a larger number of trials offers the possibility to compute system dynamics, which in turn might offer better pathways in understanding changes in development and the relations between different components (Spivey and Dale, 2006; Reddy et al., 2013). Non-linear analyses have the strength to better capture the complexity of each individual. Non-linearity underscores the observation that behaviors are not proportional to their causes (Carver and Scheier, 1998). The outcome behavior might appear chaotic and noiselike where it is in fact deterministic and predictable (Heath et al., 2000). One easily applicable method for computing non-linear system dynamics is recurrence quantification analysis (RQA). RQA quantifies aspects of the temporal evolution of a collected time series, such as its predictability, variability, or repetitiveness (Webber and Zbilut, 2007). For example, Reddy et al. (2013) analyzed infants’ force data when being picked up by their mothers and found that 3-month-olds already showed anticipatory adjustments to the approach of their mother’s arms. We applied RQA to the perception measure of the data presented above and computed the recurrence rate of shifts to the goal location. Next, we correlated the recurrence rates at the different measuring points to look at individual stability and continuity. The analyses revealed that system dynamics are stable within the individual between three measuring points, 7–12: *r* = 0.628, *p* = 0.009; 8–9: *r* = 0.413, *p* = 0.045; 9–12: *r* = 0.444, *p* = 0.05. Thus, measures that take into account non-linearity may possibly reveal reliable developmental interrelations in infants (for more examples of non-linear analyses, see Giese et al., 1996; Boker et al., 1998; Taga et al., 1999; Deffeyes et al., 2009). Furthermore, there are other non-linear analyses that could meet the obvious non-linear characteristics of development, like fractality and 1/f (for an introduction to the different non-linear measures and calculations, see Heath et al., 2000; Riley and van Orden, 2005; Holden et al., 2013). These kinds of analyses can complement traditional analyses and might eventually lead to a better understanding of children’s development. What is equally important is to run longitudinal studies when aiming at investigating developmental changes in certain abilities. The heterogeneity in individual development can tell us more about the mechanisms than a cross-sectional growth curve does (Jenni et al., 2013; Lindenberger et al., 2013).

To conclude, we presented theoretical considerations and supporting data that imply inconsistency and discontinuity of individual action and perception skills in infancy. Even though there are some precautions one could take to address this individual discontinuity, we believe that no definite conclusions can be drawn about the development of the link between action and perception in infancy. More precisely, with current methodological standards, there can be no accurate interpretation about the time when a link between action and perception is established, or which ability develops first. The nature of individual discontinuity results in the fact that some samples will show incidental correlations, while others will not. In our opinion, valid conclusions can only be achieved by applying a multi-method approach in order to better capture individual variance in development.

### Conflict of Interest Statement

The authors declare that the research was conducted in the absence of any commercial or financial relationships that could be construed as a potential conflict of interest.
